# Impact of Psychosocial Environment on Young Children’s Emotional and Behavioral Difficulties

**DOI:** 10.3390/ijerph14101278

**Published:** 2017-10-24

**Authors:** Regina Grazuleviciene, Sandra Andrusaityte, Inga Petraviciene, Birute Balseviciene

**Affiliations:** 1 Department of Environmental Sciences, Vytautas Magnus University, K. Donelaicio str. 58, 44248 Kaunas, Lithuania; sandra.andrusaityte@vdu.lt (S.A.); inga.petraviciene@vdu.lt (I.P.); 2 Department of Theoretical Psychology, Vytautas Magnus University, K. Donelaicio str. 58, 44248 Kaunas, Lithuania; birute.balseviciene@vdu.lt

**Keywords:** education level, psychosocial stress, mother-child relationships, children mental difficulties

## Abstract

*Objective:* The impact of maternal psychosocial stress on young children’s mental difficulties is unclear. This study investigated the joint effects of the socioeconomic status and parent-child relationships on emotional and behavioral difficulties in preschool children. *Methods:* The case-control study included 1416 mothers and their 4–6 year-old children pairs, living in Kaunas city, Lithuania. The parent-child relationships were measured using the Parent-Child Dysfunctional Interaction subscale. Children’s mental health difficulties were assessed by the Strengths and Difficulties Questionnaire. We used logistic regression models to indicate the strength of the associations. *Results:* Lower socioeconomic status women more often than higher ones reported pathological mother-child relations. Low education level was associated with statistically significant increase adjusted odds ratios for emotional symptoms and total behavioral difficulties. With reference to the group of better-educated mothers and normal mother-child relations, low education and pathological mother-child relations statistically significantly increased the risk of total difficulties in 4–6 year-old children; the adjusted odds ratios were 2.45; 95% CI 1.51–3.99. *Conclusions:* Pathological mother-child relations strengthened the effect of lower education on the increased risk of emotional and behavioral difficulties in preschool-age children. Measures oriented towards health behavior and psychosocial difficulties management may decrease children’s emotional and behavioral difficulties.

## 1. Introduction

Psychosocial environment in early childhood influences the health and well-being of the growing individual, and may also impact the later development of emotional and behavioral difficulties in adults’ health [1,2]. The prevalence of serious emotional and behavioral difficulties among children varies significantly depending on the study methodology, and the criteria and methods used for mental health disorder estimation, it ranges from 5% to 26% [3]. The causes of mental difficulties in childhood are numerous and inter-related. The individual and contextual factors, such as the family’s socioeconomic status (SES), parenting stress, and other environmental exposures, may influence children’s emotional and behavioral difficulties [4]. Family climate has a significant impact in predicting behavior difficulties in children. There is some evidence that the impact of stressful parenting behavior on a child’s mental health might be associated with the child’s biological pathways through stress hormone secretion [5] and genetic sensitivity [6]. Stress and unfavorable environmental exposures may trigger epigenetic changes, leading to altered metabolic pathways involved in the etiology of chronic diseases [7].

Previous studies have revealed that specific parenting stress is related to children’s emotional difficulties [8] and behavioral difficulties [9], and that lower SES is associated with increased numbers of unhealthy behaviors [10].

Although problematic behavior in children and adolescents has frequently been associated with unfavorable social environment, it is unclear to what level this relationship is affected by the mode of parent-young child relationship. Most of the above-mentioned studies were cross-sectional, used small sample sizes, and separately analyzed the impact of socioeconomic status and parenting stress on children’s mental health. Different measures were used to assess family functioning and parenting stress across numerous studies and countries, different attempts were made to control for confounding variables, and thus the influence of the psychosocial environment in the association with young children’s emotional and behavioral difficulties remains vague. 

To our knowledge, the current study is one of the first to use a large sample size of mother-child pairs in the study of the joint effect of maternal education level and mother-child relations on the risk of mental difficulties in 4–6 year-old children. This study employed psychologically valid instruments to measure dysfunctional parent-child interaction and young children’s emotional and behavioral difficulties in a large nested case-control study from Kaunas city, Lithuania, controlling for the impact of the main risk factors for mental difficulties. We hypothesized that children in low educated families are more often exposed to dysfunctional mother-child interactions, and are more likely to have emotional and behavioral difficulties.

## 2. Materials and Methods

### 2.1. Participants

The children and families represented in our study were enrolled in the Kaunas population-based pregnant women cohort study in 2007–2009, which included 4260 participants [11]. In 2012–2013, we invited 3294 mothers and their 4–6 year-old children to participate in this study. We received responses to a postal questionnaire from 1489 mothers whose residence address from the childbirth (response rate 45.2%) had not changed. This study used the similar inclusion criteria and a flow chart of the studied population as have been provided previously [12].

Because some data on children’s mental health were missing, this study included 1416 participants’ pairs. All of the parents provided written informed consents, and the study protocol was approved by the Lithuanian Bioethics Committee on 30 April 2012, resolution No. 6B-12-147. The study complies with the Declaration of Helsinki. This nested case-control study explored the relationship between mother-child interactions, SES, and the risk of emotional symptoms, conduct problems, hyperactivity, and peer problems, and the sum of the problems as the total difficulties score (TDS) in 1416 4–6 year-old children. Children mean age was 4.67 years, 49.2% of all the children participants were boys and 50.8% girls. Cases of children’s total mental difficulties (TDS) were identified in the studied population (N = 230), and were compared with the reference group of children without the TDS (N = 1186). 

### 2.2. Measurements 

Data on baseline characteristics and exposures were collected by standardized questionnaires [11,13]. The mothers reported on their residential history, family status, age, education level, SES, occupation, job during pregnancy, health behavior, chronical disease, birth outcomes, children’s development, mother-child interactions, their children’s mental health difficulties, and other covariates.

Children’s mental health difficulties were measured by the parent version of Strengths and Difficulties Questionnaire (SDQ), which is recommended for the screening mental health difficulties in a community sample among 4–16 year-old children [14]. The questionnaire was designed to assess emotional and behavioral difficulties in children. The following four subscales were used: conduct problems (e.g., “Often fights with other children and bullies them”); hyperactivity (e.g., “Constantly fidgeting or squirming”); emotional problems (e.g., “Many worries, often seems worried”); and, peer relationship problems (e.g., “Gets along better with adults than with other children. There are five items per subscale. All of the subscales are summed up to generate the total difficulties score (TDS). The participants’ responses are provided on a 3-point Likert scale that ranges from 0 (indicating strong disagreement) to 2 (indicating strong agreement), scoring 0, 1, and 2, respectively. Higher scores on the SDQ indicate more emotional and behavioral difficulties. In our sample, the mother-reported total difficulties score of the SDQ had a Cronbach’s alpha of 0.78. The internal consistency of each subscale was as follows: conduct problems: 0.62, hyperactivity: 0.64, emotional difficulties: 0.63, and peer relationship problems: 0.62. These alphas were in line with those found in previous studies [15]. Three categorical variables were created by using percentiles as cut-off points: normal—below the 85th percentile, borderline—the 85th to the 90th percentile, and mental health difficulties (pathological)—above the 90th percentile. In a stratified analysis, children’s mental health difficulties were defined by using the “normal” cut-off point (below the 85th percentile) as a reference group.

### 2.3. Exposure Assessment

The self-reported maternal education level was used as the SES indicator [16]. Maternal education level was classified as low education (10 or fewer years), medium (non-university) education, or good (university degree). In categorical analyses, the maternal education level was treated as “low” if the education was 10 or fewer years and “better if the education was more than 10 years. 

To evaluate maternal self-reported mother-child relationships, we used the Parent-Child Dysfunctional Interaction subscale (PCDI) of the Parenting Stress Index short form (S-PSI/SF) [12,17,18]. The PCDI subscale measures of the parent-child interactions index. In this study, the internal consistency of the S-PSI/SF, as measured by Cronbach’s alpha, was 0.91. Three categorical variables were created by using percentiles as cut-off points: normal—below the 85th percentile, borderline—the 85th to 90th percentile, and pathological parent-child relations—above the 90th percentile. In a multivariable analysis, parent-child interaction status was analyzed as a categorical variable with mother-child relations below the 85th percentile as the reference group. In the absence of “standard”, which could be used as a basis for establishing a cut-off point for young children in an epidemiological study of the community sample, we used 85th percentile as a borderline to assess young children mental difficulties in this epidemiological study, and assessed the prevalence of children mental difficulties in the three exposure (parent-child relations) groups: normal—scores below the 85th percentile, borderline—the 85th to 90th percentile, and pathological parent-child relations—scores above the 90th percentile. Scores above the 85th percentile are considered to be borderline clinically significant [19,20].

### 2.4. Statistical Analysis

First, in the studied population, we identified all of the children who had mental health difficulties. Subsequently, we assessed the frequency of the risk factors in case subjects and the frequency of the risk factors in the subjects of the reference group. Analyses in this study were conducted using the methods described by Jekel et al. [21]. Procedures of the univariate analysis and multivariate logistic regression models were used to determine the most reliable and robust prediction of children’s emotional and behavioral difficulties using the selected risk factors. We used chi-squared and univariate logistic regression analyses to the compare values and frequencies of the baseline characteristics by children’s emotional symptoms, conduct problems, hyperactivity and peer problems, maternal education level, and by mother-child relations of the study subjects. Mother-child relations were analyzed as a categorical variable with “normal” mother-child relations as the reference group. The covariates that were related to an increased risk of children’s emotional and behavioral difficulties and whose univariate test showed a statistically significant association (*p* < 0.05), with the outcome being treated as predictors of mental difficulties and were retained for inclusion in multivariate logistic regression analyses.

A hierarchical approach was used in the regression models to assess whether the associations between the exposure to pathological mother-child relations and lower education and 4–6 year-old children’s emotional and behavioral difficulties were affected by risk factors obtained from the analysis or those that changed the adjusted odds ratios (aOR) by 10% or more. The exposure effects were estimated as adjusted odds ratios with 95% confidence intervals (CI). Odds ratios were adjusted for individual-level covariates that could potentially affect the strength of the association: maternal age, family status, education, smoking, and the child’s sex, birth weight, siblings, and sedentary behavior.

In the stratified analysis, seeking to assess the joint effect of maternal education level and mother-child relations on the risk of total mental difficulties in 4–6 year-old children, the reference group was better-educated mothers with normal mother-child relations.

All of the statistical analyses were performed using SPSS version 20.0 (IBM Corporation, New York, NY, USA).

## 3. Results

The analyses of non-participant’s characteristics on the basis of data available from the at-birth questionnaire revealed that the demographic variables of the non-participants were not statistically significantly different from those of the participants with regard to the education level, other characteristics, and birth outcomes. 

Table 1 presents the comparison of the participants’ characteristics by children’s mental health difficulties and the chi square test for linear trend for every studied mental health difficulties associated with the increase in parent-child relations scores. The two compared groups of children with mental health difficulties (case group) and without difficulties (reference group) differed significantly concerning the some studied risk factors: children with emotional symptoms differed by secondhand smoking, maternal education level, the child’s birth order, birth weight, sedentary behavior, and mother-child relations; the children with conduct problems differed by family status and parent-child relations; children with hyperactivity differed by parent-child relations; and, children with peer problems differed significantly concerning the family status, secondhand smoking, maternal age and education level, the child’s birth order, birth weight, and parent-child relations. The chi square test for linear trend suggests that there is a relatively good correspondence between the increase in parent-child relations scores and the prevalence of all studied mental health difficulties in young children. The variables that statistically significantly differed between the comparison groups were treated as potential risk factors for children mental difficulties and were included in the multivariate analyses.

Table 2 presents the comparison of the participants’ characteristics by children’s total behavior difficulties. The two compared groups of children with total difficulties (case group) and without difficulties (reference group) differed significantly concerning the family status, maternal education level, exposure to tobacco smoke, the child’s sex, birth order, and mother-child relations. The prevalence of total difficulties was higher among children of lower educated mothers than among those of the better educated ones. In this study, 34.0% of the mothers reported borderline and pathological relations with their children. The findings suggest an increased prevalence of total difficulties of children that are exposed to parent-child borderline relations characterizing scores, and those exposed to pathological parent-child relations scores to compare with scores below the 85th percentile. Total difficulties scores below the 85th percentile had a prevalence of disorders 13.2%, the 85th to 90th percentile had a prevalence of disorders 15.9%, while the scores above the 90th percentile had the prevalence of disorders of 51.9%. Univariate logistic regression analyses showed that the prevalence of total behavioral difficulties increased linearly with higher SDQ parent-child relations scores, and the chi-squared test for trend was 48.937, *p* = 0.000 (Table 2). The analyses suggest that there is a relatively good correspondence between the increase in parent-child relations SDQ scores and the odds ratios of total behavior difficulties in young children. In the univariate analysis, tobacco smoke was associated with statistically significant increase odds ratios for total behavioral difficulties (OR 1.68; 95% CI 1.26–2.24). The variables that statistically significantly increased crude odds ratios in the univariate analysis were treated as potential predictors of children’s total behavioral difficulties and were included in the multivariate analyses. 

In the next step of analysis, using multivariate models, we investigated the associations between low maternal education, borderline and pathological mother-child relations, and children’s various mental health difficulties. The odds ratios were adjusted for selected individual-level covariates that could potentially affect the strength of the associations. Table 3 presents the prevalence of children’s emotional and behavioral difficulties (%) in the total sample and the associations between low maternal education, borderline and pathological mother-child relations, and children’s various psychological problems, as adjusted odds ratios. The overall prevalence rates of emotional and behavioral difficulties among children aged 4 through to 6 years were 16.2% for total difficulties and 17.3%—for emotional symptoms. In the multivariate model, the odds ratios of the studied mental health difficulties tended to be higher for children with lower maternal education; however, statistically significant increased odds ratios were found only for emotional symptoms (by 43% (aOR 1.43; 95% CI 1.00–2.05)) and for total difficulties (by 44% (aOR 1.44; 95% CI 1.02–2.08)). Borderline and pathological mother-child relations increased the odds ratios of children’s mental health difficulties. Statistically significantly higher odds ratios were found for emotional symptoms, conduct problems, peer problems, and for total difficulties. These associations remained robust after adjustment for the marital status, education, and smoking, age at childbirth, the child’s sex, birth weight, siblings, and sedentary behavior. Based on this evidence, we concluded that lower maternal education and borderline and pathological mother-child relations were independent risk factors for children’s mental health difficulties. Our findings showed that the unfavorable environment in early life (such as low socio-economic status associated with low maternal education) and exposure to tobacco smoke could increase the risk of emotional and behavioral difficulties later in life.

To study the combined effects of low maternal education and dysfunctional mother-child relations, we in stratified models simultaneously evaluated the risks associated with mother-child relations, education level, and the risks of emotional and behavioral difficulties among 1416 4–6 year-old children (Table 4). The prevalence of total difficulties in children of better-educated mothers with normal parent-child relations was 12.4%, as compared to 17.0% in the group of low educated mothers with normal parent-child relations. Borderline and pathological parent-child relations significantly increased the prevalence rate of total difficulties in both groups—to 19.6% and 29.5%, accordingly. With reference to the group of a better education level and normal mother-child relations, borderline and pathological mother-child relations by more than two-fold (aOR 2.11) increased the risk of emotional difficulties in better-educated women’s children, while in lower educated mothers’ children, the risk increased by 2.65 times (aOR 2.65; 95% CI 1.65–4.28). Similar results were observed for conduct problems, peer problems, and total difficulties. Among 4–6 year-old children of low educated mothers with borderline and pathological mother-child relations, the risk of total difficulties was by 2.45-fold higher (aOR 2.45; 95% CI 1.51–3.99). The results showed that dysfunctional mother-child relations strengthened the effect of lower education on the increased risk of emotional and behavioral difficulties in preschool-age children. These findings were robust after adjustment for marital status, smoking, age, and the child’s sex, birth weight, siblings, and sedentary behavior.

## 4. Discussion

Prior studies have mostly separately studied the impact of family climate and SES on emotional and behavioral difficulties in school-age children and adolescents. To our knowledge, this study was one of the first to examine the association between pathological mother-child relations associated with SES and emotional and behavioral difficulties in 4–6 year-old children by using psychologically valid instruments in a large population-based sample and by controlling the impact of potential confounding variables. Therefore, comparisons of our findings with observations from similar studies were limited. This study found evidence that the psychosocial factor—pathological mother-child relations—strengthened the effect of lower maternal education on the increasing risk of emotional and behavioral difficulties in 4–6 year-old children. Our study supports the hypothesis that children in low educated families are more often exposed to dysfunctional mother-child interaction and are more likely to have emotional and behavioral difficulties. Moreover, children with behavioral difficulties were more often exposed to unhealthy modifiable behaviors, such as tobacco smoke and dysfunctional mother-child relations. These findings suggest that measures oriented towards psychosocial stress management and health behavior should be encouraged among parents in order to decrease the risk of emotional and behavioral difficulties in their children. 

In the total sample of 4–6 year-old Kaunas children, the prevalence of total difficulties was 16.2%, and it was similar to that reported in the European study that used the SDQ for screening mental health difficulties among 6–11 year-old children (15.5%) [22]. The prevalence of mental health difficulties among children and adolescents in Germany is between 10% and 18% [23]. The overall prevalence rates of mental health difficulties among Chinese children were 7.6%, and for total difficulties were 10.9% [24]. The similar prevalence of total mental difficulties reported in the European study that used the SDQ for screening mental health difficulties among 6–11 year-old children, suggest that in this study, used scores of the 85th percentile may be a cut-off score in epidemiological studies for screening young children mental difficulties.

The study of the associations between the participants’ characteristics and children’s total difficulties confirmed the following predictors of young children’s mental health difficulties: a single mother, lower maternal education, and exposure to tobacco smoke, pathological mother-child relations, the child’s male sex, and being the first child. Low maternal education level increased the children’s risk of total difficulties by 44% (aOR 1.44; 95% CI 1.02–2.08), and borderline and pathological mother-child relation—by 69% (aOR 1.69; 95% CI 1.24–2.31).

These results are consistent with the increasing body of evidence that multiple psychosocial and biological factors are associated with children’s mental health, and children with many risk factors are at a higher risk [25]. Parenting practice and the parents’ separation or divorce are significant risk factors [26], other factors influencing the children’s mental well-being are mother’s socioeconomic status, as well as other household characteristics [10]. Unfavorable prenatal and early-life environmental factors, lower social competence [1], and social disadvantage are considered to be potential risk factors for mental health disorders in childhood [27]. Our findings on an adverse impact of tobacco smoke on young children’s mental health are in line with the results of the studies reporting that household smoking can lead to behavioral difficulties in children, particularly emotional and behavioral disorders [28].

In this study, the prevalence of total difficulties in 4–6 year-old children was associated with SES: it was significantly higher in children born to mothers with lower education levels than in children of better-educated mothers. Recent studies have presented similar results showing that lower maternal education was significantly related to higher SDQ scores and much stronger associations with mental health disorders of children [10,29].

The results of this study showed that the prevalence of total difficulties in 4–6 year-old children was also associated with mother-child relations: the prevalence was significantly higher of children of mothers with dysfunctional mother-child interaction than in mother-child pairs with normal relations. In this study, 34.0% of the mothers reported borderline or pathological relations with their children. With reference to normal relations, borderline and pathological relations were associated with statistically significantly increased emotional symptoms, conduct problems, and peer problems. Adjusted odds ratios for total difficulties in the presence of borderline and pathological relations were 1.69, 95% CI 1.24–2.31. Our significantly higher odds ratios for pathological mother-child relationships suggest a relationship between the psychosocial environment and children’s emotional and behavioral difficulties. Previous studies that analyzed risk factors predictive of the behavioral disorders, not studied combined of SES and mother-child relationships effects, demonstrated that higher levels of parenting stress were related to higher levels of children’s emotional and behavioral symptoms [27,30]. This study of the combined effects of maternal education and dysfunctional relations on children’s mental health difficulties revealed that the associations varied depending on both SES and parent-child relations. With reference to better maternal education and normal mother-child relations, low education and borderline and pathological mother-child relations were associated with statistically significantly by more than two-fold increased odds ratios for emotional symptoms, conduct problems, and peer problems. Among 4–6 year-old children of lower educated mothers with borderline and pathological mother-child relations, the risk of total difficulties was by 2.45-fold higher (aOR 2.45; 95% CI 1.51–3.99). The results showed that dysfunctional mother-child relations strengthened the effect of lower education on the increased risk of emotional and behavioral difficulties in preschool-age children. These findings are in line with those of a Spanish study indicating that higher levels of mothers’ mental health difficulties and low SES increased the risk of 4–14-year-old children exhibiting behavioral difficulties, being hyperactive, or demonstrating antisocial behavior (10). However, in a small study (N = 125) conducted in the USA, no evidence was found to support the premise that parental behavior mediates the association between parental stress and behavior outcomes in 5 year-old children [4].

This study presented the evidence for the strength of the disadvantageous effects of low SES and pathological parent-child relations on early psychosocial development. Therefore, good mother–child relations can promote the development of healthy emotions and behavior regulation in their children.

Our study has advantages over the previous similar ones because of its case-control design, large sample size, and employed psychologically valid instruments to measure the mother-child interactions and children’s mental health difficulties, and the use of multivariate analysis. Using individual data, we for the first time estimated the effects of low SES on the prevalence of pathological mother-child relations and jointly assessed the associations for the groups of children born to mothers with higher and lower education levels and with normal and pathological mother-child relations.

The limitations of our study include the omission of some aspects of family functioning. We cannot exclude that residual non-controlled confounding variables might have affected the observed higher risk for mental health difficulties in children of mothers with lower SES because of a less healthy environment. Other limitations include the use of subjective self-report measures of dysfunctional mother-child relations, which may have biased measures, and studying of parent-child relations data from a sample that was predominantly mothers. However, in this study, we controlled for the main variables that might have confounded the studied associations of young children’s mental health difficulties. Our adjusted associations suggest that low SES and pathological mother-child relations were determinants that were associated with young children’s mental health difficulties.

## 5. Conclusions

The findings of this study strengthen the evidence that being in a family with a low SES is more often associated with exposure to dysfunctional mother-child interaction and other modifiable health behavior that contributes to an increased risk of emotional and behavioral difficulties in young children. The effect of low maternal education on children’s mental health depended on the quality of mother-child relationships. Our results suggest that children with total difficulties were more often exposed to tobacco smoke and dysfunctional mother-child relations, lived with low educated, single mothers, and were the first child. The findings of the study provide evidence that measures oriented towards health behavior and psychosocial difficulties management may decrease children’s emotional and behavioral difficulties. The key implication is the importance of screening for behavioral difficulties in low-SES families. Future studies might focus on effects of changes in the early-life environment and the modification of predictors related to mental health in children. 

## Figures and Tables

**Table 1:** Prevalence of mental health difficulties in preschool age children.

Risk Factors	Control (No Emotional Symptoms)	Cases (Yes Emotional Symptoms) N = 245	Control (No Conduct Problems)	Cases (Yes Conduct Problems) N = 195	Control (No Hyperactivity) N (%)	Cases (Yes Hyperactivity) N = 215	Control (No Peer Problems)	Cases (Yes Peer Problems) N = 202
Family status								
Both parents	1036 (83.3)	208 (16.7)	1085 (87.2)	159 (12.8)	1067 (85.8)	177 (14.2)	1074 (86.3)	170 (13.7)
Single mother	135 (78.5)	37 (21.5)	136 (79.1)	36 (20.9) *	134 (77.9)	38 (22.1) *	140 (81.4)	32 (18.7)
Smoking during pregnancy								
No	1091 (83.2)	220 (16.8)	1135 (86.6)	176 (13.4)	1116 (85.1)	195 (14.9)	1123 (85.7)	188 (14.3)
Yes	80 (76.2)	25 (23.8)	86 (81.9)	19 (18.1)	85 (81.0)	20 (19.0)	91 (86.7)	14 (13.3)
Secondhand smoking								
No	769 (84.5)	141 (15.5)	795 (87.4)	115 (12.6)	791 (86.9)	119 (13.1)	786 (86.4)	124 (13.6)
Yes	402 (79.4)	104 (20.6) *	426 (84.2)	80 (15.8)	410 (81.0)	96 (19.0) *	428 (84.6)	78 (15.4)
Maternal age at childbirth								
≤30 years	783 (82.2)	169 (17.8)	816 (85.7)	136 (14.3)	793 (83.3)	159 (16.7)	824 (86.6)	128 (13.4)
>30 years	388 (83.6)	76 (16.4)	405 (87.3)	59 (12.7)	408 (87.9)	56 (12.1) *	390 (84.1)	74 (15.9)
Maternal education								
Low	216 (76.6)	66 (23.4) *	233 (82.6)	49 (17.4)	223 (79.1)	59 (20.9) *	232 (82.3)	50 (17.7)
Medium	220 (82.4)	47 (17.6)	234 (87.6)	33 (12.4)	228 (85.4)	39 (14.6)	227 (85.0)	40 (15.0)
Good	735 (84.8)	132 (15.2)	754 (87.0)	113 (13.0)	750 (86.5)	117 (13.5)	755 (87.1)	112 (12.9)
Child’s birth weight								
≤2500 g	60 (74.1)	21 (25.9) *	70 (86.4)	11 (13.6)	58 (71.6)	23 (28.4) *	68 (84.0)	13 (16.0)
>2500 g	1111 (83.2)	224 (16.8)	1151 (86.2)	184 (13.8)	1143 (85.6)	192 (14.4)	1146 (85.8)	189 (14.2)
Child’s sex								
Male	577 (82.9)	119 (17.1)	589 (84.6)	107 (15.4)	571 (82.0)	125 (18.0) *	586 (84.2)	110 (15.8)
Female	594 (82.5)	126 (17.5)	632 (87.8)	88 (12.2)	630 (87.5)	90 (12.5)	628 (87.2)	92 (12.8)
Sedentary behavior								
≤3 h per day	957 (84.0)	182 (16.0)	987 (86.7)	152 (13.3)	972 (85.3)	167 (14.7)	987 (86.7)	52 (13.3)
>3 h per day	214 (77.3)	63 (22.7) *	234 (84.5)	43 (15.5)	229 (82.7)	48 (17.3)	227 (81.9)	50 (18.1)
Child’s BMI								
<18 kg/m^2^	1082 (82.7)	226 (17.3)	1128 (86.2)	180 (13.8)	1114 (85.2)	194 (14.8)	1123 (85.9)	185 (14.1)
≥18 kg/m^2^	89 (82.4)	19 (17.6)	93 (86.1)	15 (13.9)	87 (80.6)	21 (19.4)	91 (84.3)	17 (15.7)
Birth order								
First child	622 (79.1)	164 (20.9) *	677 (86.1)	109 (13.9)	660 (84.0)	126 (16.0)	666 (84.7)	120 (15.3)
2nd and later	549 (87.1)	81 (12.9)	544 (86.3)	86 (13.7)	541 (85.9)	89 (14.1)	548 (87.0)	82 (13.0)
Parent-child relations *								
Normal	814 (87.1)	121 (12.9)	833 (89.1)	102 (10.9)	796 (85.1)	139 (14.9)	817 (87.4)	118 (12.6)
Borderline	310 (78.5)	85 (21.5) ^a^	327 (82.8)	68 (17.2) ^b^	346 (87.6)	49 (12.4) ^c^	346 (87.6)	49 (12.4) ^d^
Pathological	47 (54.7)	39 (45.3) ^a^	61 (70.9)	25 (29.1) ^b^	59 (68.6)	27 (21.4) ^c^	51 (59.3)	35 (40.7) ^d^

* *p* (*p*-value) < 0.05. ^a^ Chi square test for linear trend 56.569, *p* = 0.000; ^b^ Chi square test for linear trend 26.659, *p* = 0.000; ^c^ Chi square test for linear trend 4.081, *p* = 0.043; ^d^ Chi square test for linear trend 22.580, *p* = 0.000.

**Table 2:** Prevalence of risk factors for children’s total behavioral difficulties (TBD), crude odds ratios, and their 95% confidence intervals (CI).

Risk Factors for TBD	Cases (Yes TBD N = 230) N (%)	Controls (No TBD N = 1186) N (%)	Crude Odds Ratios (95% CI)
Family status *			
Both parents	186 (15.0)	1058 (85.0)	1
Single mother	44 (25.6)	128 (74.4)	1.96 (1.34–2.85)
Smoking during pregnancy			
No	207 (15.8)	1104 (84.2)	1
Yes	23 (21.9)	82 (78.1)	1.50 (0.92–2.43)
Secondhand smoking *			
No	124 (13.6)	786 (86.4)	1
Yes	106 (20.9)	400 (79.1)	1.68 (1.26–2.24)
Maternal age at childbirth			
≤30 years	165 (17.3)	787 (82.7)	1.29 (0.94–1.76)
>30 years	65 (14.0)	399 (86.0)	1
Maternal education *			
Low	64 (22.7)	218 (77.3)	1.90 (1.35–2.67)
Medium	50 (18.7)	217 (81.3)	1.49 (1.04–2.15)
Good	116 (13.4)	751 (86.6)	1
Child’s birth weight			
≤2500 g	17 (21.0)	64 (79.0)	1.40 (0.80–2.44)
>2500 g	213 (16.0)	1122 (84.0)	1
Child’s sex *			
Male	131 (18.8)	565 (81.2)	1.46 (1.10–1.94)
Female	99 (13.8)	621 (86.2)	1
Sedentary behavior			
≤3 h per day	177 (15.5)	962 (84.5)	1
>3 h per day	53 (19.1)	224 (80.9)	1.29 (0.92–1.81)
Child’s BMI			
<18 kg/m^2^	211 (16.1)	1097 (83.9)	1
≥18 kg/m^2^	19 (17.6)	89 (82.4)	1.11 (0.66–1.86)
Birth order *			
First child	150 (19.1)	636 (80.9)	1.62 (1.21–2.18)
2nd and later	80 (12.7)	550 (87.3)	1
Parent-child relations *^,a^			
Normal (<85th percentile)	123 (13.2)	812 (86.8)	1
Borderline (85th to 90th percentile)	63 (15.9)	332 (84.1)	1.25 (0.90–1.74)
Pathological (>90th percentile)	44 (51.2)	42 (48.8)	6.92 (4.35–10.99)

* *p* < 0.05. ^a^ Chi square test for linear trend 48.937, *p* = 0.000.

**Table 3:** Associations between low maternal education, borderline/pathological mother-child relations, and children’s mental health difficulties as adjusted odds ratios and their 95% confidence intervals (CI).

Children Mental Health	Cases (Problem) N (%)	Maternal Low Education Adjusted ^†^ Odds Ratios (95% CI)	Borderline/Pathological Mother-Child Relations Adjusted ^††^ Odds Ratios (95% CI)
No problem (referent group)		Referent: higher education-1	Referent: normal relations-1
Yes emotional symptoms	245 (17.3)	1.43 * (1.00–2.05)	2.06 * (1.53–2.77)
Yes conduct problems	195 (13.8)	1.32 (0.89–1.96)	1.83 * (1.32–2.54)
Yes hyperactivity	215 (15.2)	1.31 (0.89–1.92)	1.04 (0.75–1.44)
Yes peer problems	202 (14.3)	1.36 (0.92–2.00)	1.47 * (1.06–2.02)
Yes total difficulties	230 (16.2)	1.44 * (1.02–2.08)	1.69 * (1.24–2.31)

* *p* < 0.05. **^†^** Adjusted for: marital status, smoking, age at childbirth, the child’s sex, birth weight, siblings, and sedentary behavior. **^††^** Adjusted for: marital status, education, smoking, age at childbirth, the child’s sex, birth weight, siblings, and sedentary behavior.

**Table 4:** Joint effects of maternal education and dysfunctional relations on children mental health difficulties (the referent group of stratified analysis: higher education and normal parent-child relations).

Children Mental Health Difficulties	Cases, N (%)	Controls, N (%)	Adjusted ^†^ OR (95% CI)
Emotional Symptoms ^††,a^			
Good education & Normal parent-child relations	95 (12.1)	687 (87.9)	Referent 1
Good education & Borderline and pathological parent-child relations	84 (23.9)	268 (76.1)	2.11 (1.49–2.97)
Low education & Normal parent-child relations	26 (17.0)	127 (83.0)	1.38 (0.83–4.28)
Low education & Borderline and pathological parent-child relations	40 (31.0)	89 (69.0)	2.65 (1.65–4.28)
Conduct Problems ^††,b^			
Good education & Normal parent-child relations	83 (10.6)	699 (89.4)	Referent 1
Good education & Borderline and pathological parent-child relations	63 (17.9)	289 (82.1)	1.67 (1.14–2.43)
Low education & Normal parent-child relations	19 (12.4)	134 (87.6)	1.01 (0.57–1.81)
Low education & Borderline and pathological parent-child relations	30 (23.3)	99 (76.7)	2.47 (1.49–4.11)
Hyperactivity ^††,c^			
Good education & Normal parent-child relations	105 (13.4)	677 (86.6)	Referent 1
Good education & Borderline and pathological parent-child relations	51 (14.5)	301 (85.5)	1.03 (0.70–1.51)
Low education & Normal parent-child relations	34 (22.2)	119 (77.8)	1.29 (0.79–2.12)
Low education & Borderline and pathological parent-child relations	25 (19.4)	104 (80.6)	1.36 (0.80–2.31)
Peer problems ^††,d^			
Good education & Normal parent-child relations	97 (12.4)	685 (87.6)	Referent 1
Good education & Borderline and pathological parent-child relations	55 (15.6)	297 (84.4)	1.37 (0.95–1.99)
Low education & Normal parent-child relations	21 (13.7)	132 (86.3)	1.15 (0.66–1.97)
Low education & Borderline and pathological parent-child relations	29 (22.5)	100 (77.5)	2.04 (1.22–3.43)
Total difficulties ^††,e^			
Good education & Normal parent-child relations	97 (12.4)	685 (87.6)	Referent 1
Good education & Borderline and pathological parent-child relations	69 (19.6)	283 (80.4)	1.58 (1.10–2.27)
Low education & Normal parent-child relations	26 (17.0)	127 (83.0)	1.19 (0.71–2.01)
Low education & Borderline and pathological parent-child relations	38 (29.5)	91 (70.5)	2.45 (1.51–3.99)

**^†^** Adjusted for: marital status, smoking, age, and the child’s sex, birth weight, siblings, and sedentary behavior. ^††^ Total number of mental health difficulties cases: ^a^ Emotional Symptoms N = 245, ^b^ Conduct Problems N = 195, ^c^ Hyperactivity N = 215, ^d^ Peer problems N = 202, ^e^ Total difficulties N = 230.
